# Systemic veterinary drugs for control of the common bed bug, *Cimex* *lectularius*, in poultry farms

**DOI:** 10.1186/s13071-022-05555-6

**Published:** 2022-11-17

**Authors:** Maria A. González-Morales, Andrea E. Thomson, Olivia A. Petritz, Rocio Crespo, Ahmed Haija, Richard G. Santangelo, Coby Schal

**Affiliations:** 1 Department of Entomology and Plant Pathology, North Carolina State University, Raleigh, NC USA; 2 Department of Clinical Sciences, College of Veterinary Medicine, North Carolina State University, Raleigh, NC USA; 3 Department of Population Health and Pathobiology, College of Veterinary Medicine, North Carolina State University, Raleigh, NC USA

**Keywords:** *Cimexlectularius*, Fluralaner, Ivermectin, Systemics, Poultry, Chicken

## Abstract

**Background:**

The common bed bug, *Cimex* *lectularius* L., is a hematophagous ectoparasite that was a common pest in poultry farms through the 1960s. Dichlorodiphenyltrichloroethane (DDT) and organophosphates eradicated most infestations, but concurrent with their global resurgence as human ectoparasites, infestations of bed bugs have been reappearing in poultry farms. Although the impact of bed bugs on chicken health has not been quantified, frequent biting and blood-feeding are expected to cause stress, infections and even anemia in birds. Bed bug control options are limited due to the sensitive nature of the poultry environment, limited products labeled for bed bug control and resistance of bed bug populations to a broad spectrum of active ingredients. Veterinary drugs are commonly used to control endo- and ectoparasites in animals. In this study, we evaluated the effects of two common veterinary drugs on bed bugs by treating the host with systemic antiparasitic drugs.

**Methods:**

We conducted dose–response studies of ivermectin and fluralaner against several bed bug strains using a membrane feeding system. Also, different doses of these drugs were given to chickens and two delivery methods (topical treatment and ingestion) were used to evaluate the efficacy of ivermectin and fluralaner on bed bug mortality.

**Results:**

Using an artificial feeding system, both ivermectin and fluralaner caused high mortality in insecticide-susceptible bed bugs, and fluralaner was found to be effective on pyrethroid- and fipronil-resistant bed bugs. Ivermectin was ineffective in chickens either by the topical treatment or ingestion, whereas bed bugs that fed on chickens which had ingested fluralaner suffered high mortality when feeding on these chickens for up to 28 days post treatment.

**Conclusions:**

These findings suggest that systemic ectoparasitic drugs have great potential for practical use to control bed bug infestations in poultry farms. These findings also demonstrate the efficacy of fluralaner (and potentially other isoxazolines) as a potent new active ingredient for bed bug control.

**Graphical Abstract:**

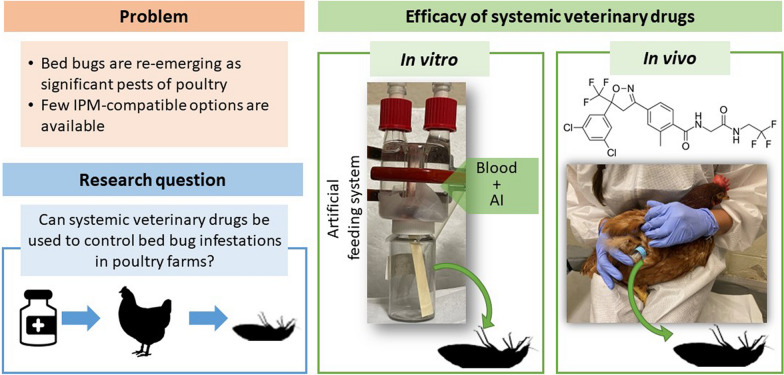

## Background

The common bed bug (*Cimex* *lectularius* L.) is an obligate hematophagous ectoparasite that feeds on humans. However, bed bugs opportunistically parasitize other animals, including birds and bats [1]. Infestations of bed bugs in poultry farms were reported as early as the 1940s in North America [2] and Europe [3]. In the USA, bed bugs were reported as major pests in poultry in 1985 [4].

Bed bugs are wingless, nocturnal, cryptic insects that have limited dispersal capabilities; thus, it is likely that the introduction of bed bugs to poultry facilities is human-mediated either though the supply chain or by farm workers [4]. Although the effects of bed bugs on poultry health are understudied, it is reasonable to expect, as with other blood-feeding ectoparasites, that bed bug infestations would cause pruritus, feather pecking, restlessness, anemia, secondary infections and an overall decrease in poultry health and production [5, 6].

Bed bug infestations were largely eradicated from the poultry industry during the late 1940s with the use of dichlorodiphenyltrichloroethane (DDT) and organophosphates [3]. Today, pyrethroids are the primary class of insecticides used in the poultry industry to control bed bug populations, along with some organophosphates, spinosyns and neonicotinoids. Pyrethroid resistance is widespread in bed bug populations worldwide [7], and target-site resistance (knockdown resistance [*kdr*] mutations) has dramatically increased in bed bug populations in the last decade [8]. Therefore, highly resistant bed bugs are expected to be introduced into poultry farms. Limited availability of insecticides and resistance to the most commonly used insecticides appear to be major constraints of bed bug control in poultry farms. Some dust formulations of inorganic insecticides are also available, but their efficacy in the challenging poultry environment has been inconsistent [9].

Xenointoxication, the systemic treatment of hosts to kill parasites, is widely used in human [10, 11] and veterinary medicine to control endoparasites (e.g. mosquito-borne pathogens) and ectoparasites such as mosquitoes, ticks, mites and fleas [12]. This strategy has been broadly accepted for use in pets and companion animals. Studies that supplemented blood with insecticides in membrane-based artificial feeders have demonstrated considerable mortality in the common bed bug; effective active ingredients (AIs) include conventional insecticides, such as abamectin and fipronil [13], and antiparasitic drugs, such as ivermectin, moxidectin [14] and fluralaner [15]. In vivo xenointoxication was also shown to be effective on bed bugs that were fed on ivermectin-treated mice [16] and rabbits [17].

Ivermectin, first introduced as an antiparasitic drug in 1981 [12, 18], is considered to be safe and effective, and is commonly used in humans to control parasitic infections transmitted by mosquitoes (e.g. lymphatic filariasis), reduce malaria transmission and treat scabies, onchocerciasis and myasis [19]. The wide variety of uses of ivermectin and other avermectins include companion animals (dogs and cats) and livestock (cattle, horses, sheep and swine) to control endoparasites such as heartworm and roundworm and ectoparasites, which include mites, fleas and ticks [19]. Currently, prescription-based ivermectin formulations are available for use in poultry with appropriate post-treatment withdrawal periods [20].

Fluralaner is a relatively new drug. It was introduced in 2014 as a flea treatment for dogs and in 2019 for cats. Fluralaner belongs to the isoxazolines class of insecticides that includes only parasiticide compounds. Several studies have evaluated the efficacy of fluralaner administered to hens to control the poultry red mite, *Dermanyssus* *gallinae* [21, 22], and the northern fowl mite, *Ornithonyssus* *sylviarum* [23]. In Europe, fluralaner is approved for use in chickens to control mites. However, currently there are no formulations of fluralaner approved for use in any poultry species in the USA.

The goal of this project was to evaluate systemic veterinary drugs in chickens as a potentially efficient way to suppress or even eradicate bed bug infestations from poultry farms. To this end, we conducted dose–response studies with ivermectin and fluralaner using a membrane feeding system in which blood could be dosed with precise concentrations of the AIs. We then transitioned to chicken flocks and tested the efficacy of these drugs against bed bugs that fed on treated chickens.

## Methods

### Experimental insects and rearing procedures

The Harold Harlan (Harlan) strain of *C.* *lectularius* was collected at Fort Dix, New Jersey (USA) in 1973 and maintained on a human host until 2008. Between 2008 and 2021, the Harlan strain was maintained in our laboratory on defibrinated rabbit blood, and since 2021 on human blood. Since its collection, the Harlan population has not been challenged with insecticides and, therefore, it was used in this study as an insecticide-susceptible reference strain. Five other more recently field-collected strains were assayed in the in vitro dose–response feeding experiments. Healthy young adult males were used throughout all experiments. Bed bug health was assessed by the shape of the antennae and legs and general coordination of the insects.

### Bed bug rearing and artificial feeding system

**Fig. 1:**
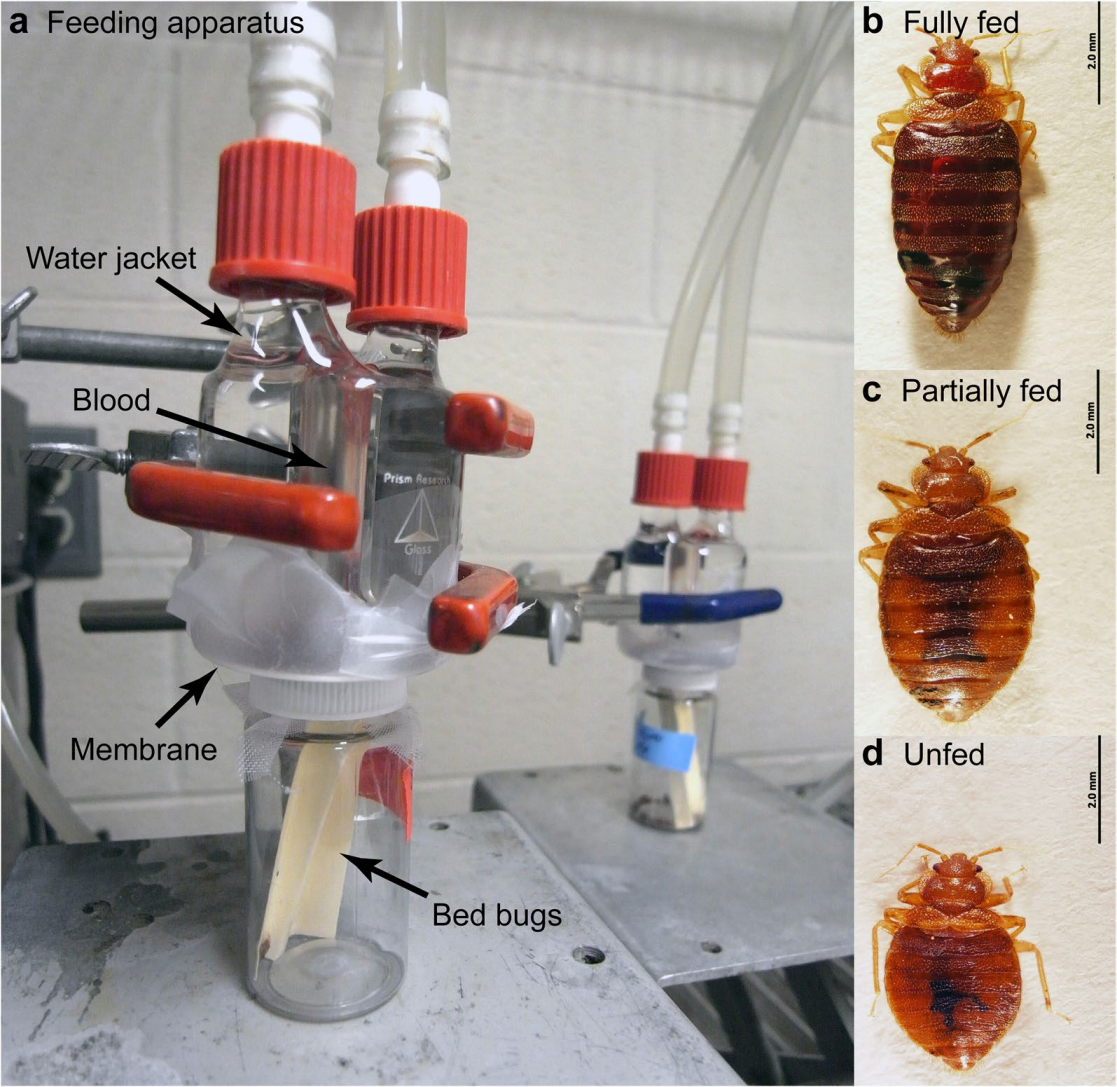
**a** Artificial feeding system used to feed bed bugs insecticide-supplemented human blood. Blood was placed into the internal chamber of a custom-fabricated glass feeder heated with a circulating water bath and held in place by plant grafting tape. Bed bugs were placed in PET plastic vials containing cardboard to provide harborage and capped with plankton screen through which bed bugs could feed. **b**–**c** Only fully engorged individuals (**b**, determined visually) were retained, while partially fed (**c**) and unfed (**d**) adult males were discarded

Bed bug colonies were reared in 118-cm^3^ plastic jars containing cardstock paper substrate for harborage and capped with plankton netting (BioQuip Products, Rancho Dominguez, CA, USA) to enable aeration and feeding. Bed bugs were maintained at 25 °C, 50 ± 5% relative humidity and a photoperiod of 12:12 (light:dark) h, and fed weekly on human blood delivered through an artificial feeding system. Heparinized human blood was supplied by the American Red Cross (IRB #00000288 and protocol #2018-026). The artificial feeding system was modified after Montes et al. [24] and Sierras and Schal [13]. It was housed in an North Carolina State University (NCSU)-approved BSL-2 facility (Biological Use Authorization #2020-09-836) and consisted of custom-fabricated water-jacketed glass feeders (Fig. 1a), each with an internal blood chamber within a circulating water chamber connected to a circulating water bath heated to maintain blood near human skin temperature (approx. 32 °C). Although each blood chamber had a 4-ml capacity, we used 2 ml, which was sufficient to eliminate air bubbles from the blood chamber. Plant grafting tape (A.M. Leonard Horticultural Tool and Supply Co., Piqua, OH, USA) was stretched across the bottom of the feeder and served to hold the blood within each feeder and to function as a membrane through which bed bugs could feed. Several feeders were connected in series to the water circulator, which allowed multiple colonies to be fed concurrently.

### Pyrethroid resistance

Deltamethrin (98.9% purity; Chem Services, West Chester, PA, USA) was used in a dose–response study with the Harlan strain to estimate the dose (concentration) that killed 99% of the population (lethal dose 99% [LD_99_]), which we then used as a diagnostic dose on the field-collected strains. Adult male bed bugs, 4 days post-feeding, were placed in plastic Petri dishes (diameter: 60 mm; Thermo Fisher Scientific, Waltham, MA, USA) lined with filter paper (Whatman No. 1; Millipore-Sigma, Allentown, PA, USA). Bed bugs were briefly (< 10 s) anesthetized with CO_2_, deltamethrin (in acetone) was applied topically with a manual microapplicator (Hamilton Co., Reno, NV, USA) equipped with a 25-µl glass syringe (Hamilton Co.) that delivered 0.5 µl of solution to the ventral thorax of each insect. We used eight doses of between 0 (acetone control) and 10 ng and at least 25 adult male bed bugs per dose, for a total of approximately 240 bed bugs. Mortality was assessed every 24 h for 48 h by gently touching individual bed bugs with featherweight entomological forceps, categorizing them as alive (coordinated avoidance movement) or dead (no response or unable to right themselves after touching with forceps).

### Birds

A flock of 30 Rhode Island Red hens (*Gallus* *gallus* *domesticus*; body weight range: 1.9–2.8 kg; average weight: 2.4 kg) ranging in age from 2 to 3 years were used in this study. The birds were maintained under a photoperiod of 12:12 (light:dark) h and housed as a flock in a climate-controlled facility (15.6 m^2^) on a wood shaving substrate. Hens were obtained from a private breeder and fed an 18% protein layer diet with free access to water. The flock was monitored by veterinarians to ensure optimal health based on serial physical examinations, serial packed cell volumes via microhematocrit tube and centrifugation, serial total solids via refractometer and serial biochemical panels (VetScan Avian/Reptile Profile Plus; Abaxis Inc., Union City, CA, USA). All study procedures were approved by the NCSU Institutional Animal Care and Use Committee (IACUC #21-152).

### In vitro feeding assays using artificial feeders

To deliver each AI in blood, technical grade fluralaner (≥ 98%; Cayman Chemical, Ann Arbor, MI, USA) and ivermectin (≥ 98%; Millipore-Sigma) were dissolved in dimethyl sulfoxide (DMSO) to make stock solutions of 10 mg AI/ml DMSO, from which we made serial dilutions in DMSO to achieve the desired concentrations. A 2-µl aliquot of each AI in DMSO solution was then added to 1.998 ml of blood (0.1% DMSO final concentration in blood) just before the assay commenced.

Healthy adult male bed bugs were collected weekly from the colony and used 5–7 days post feeding on human blood (Fig. 1d). Therefore, although the bed bugs used in these assays were of unknown ages, they were generally within 2 weeks of adult emergence. For each replicate, 10 males were placed into clear 20-ml PET plastic containers containing cardboard inserts for harborage and capped with plankton netting to allow feeding. Each replicate of 10 males was given 15 min to feed on human blood, and only fully engorged individuals were retained for further study (Fig. 1b). Replete bed bugs were transferred to individual wells of 24-well cell culture plates (Corning Inc., Corning, NY, USA) containing a tightly fitting filter paper circle at the bottom of each well. Mortality was assessed every 24 h for up to 7 days post feeding. We conducted three replicates (total *n* = 30 bed bugs) for each insecticide concentration, as well as two control groups that consisted of human blood alone and human blood plus 0.1% DMSO, respectively. Adult male bed bugs from six unique field-collected strains were used.

### In vivo feeding assays with chickens

**Fig. 2:**
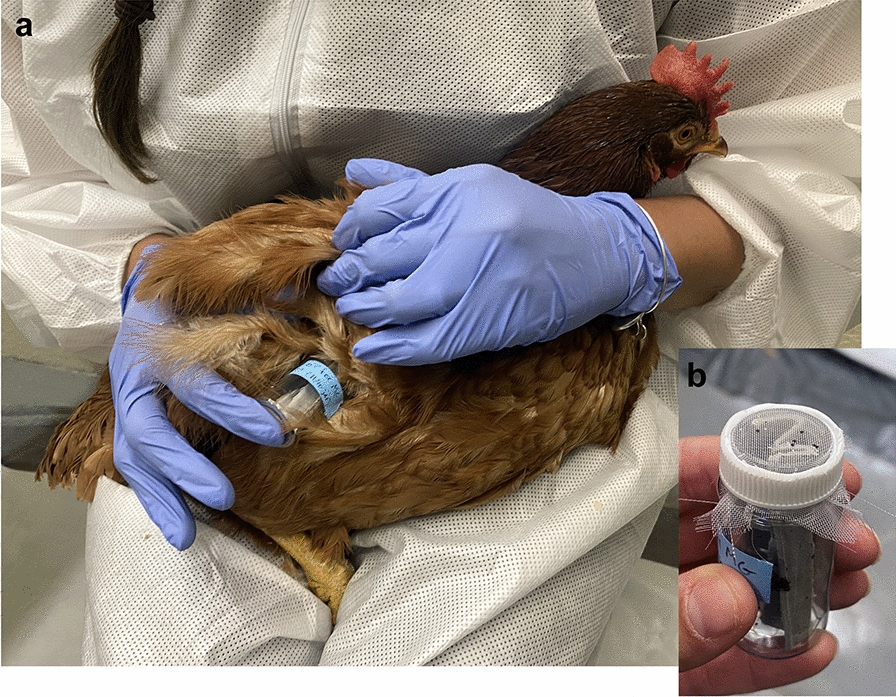
Feeding bed bugs on chickens. **a** Birds were held on the lap of a researcher. **b** A plastic vial containing a cardboard harborage and up to 15 adult male bed bugs. The cardboard also served as a ramp that provided access to a plankton screen cap through which bed bugs could feed on the bird’s lateral inguinal region. Each group of bed bugs was allowed 10 min to feed

Harlan strain adult male bed bugs (15 insects per replicate; Fig. 1d), 5–7 days post feeding on human blood, were placed into clear 20-ml PET plastic containers. Each chicken was restrained by gently placing the bird on the lap of one of the researchers and then wrapping it in a towel, as needed. The plankton netting of the bed bug container (Fig. 2b) was gently placed on the lateral inguinal area of the chicken, and bed bugs were allowed to feed for 10 min (Fig. 2a). As in the in vitro assays, fully engorged bed bugs were transferred individually to each well of 24-well cell culture plates and maintained in an incubator at rearing conditions. Each chicken was exposed to a single bed bug replicate (maximum 15 bed bugs) feeding per day, and alternate sides of the bird were used in subsequent feedings to avoid unnecessary irritation.

#### Ivermectin

Twelve chickens from the flock of 30 birds were randomly selected and divided into control (*n* = 4) and treatment (*n* = 8) groups, respectively. Ivermectin (Ivermax 1% sterile solution; Aspen Veterinary Resources, Liberty, MO, USA) was administered via subcutaneous injection in two birds, and the dose was adjusted to the weight of each bird (0.2 mg/kg body mass). The same dose of ivermectin (0.2 mg/kg) was administered to six birds via oral gavage using a needleless syringe. The dose of 0.2 mg/kg was based on Cirak et al. [25]. The remaining four chickens were assigned to a control group that received a water gavage. Each chicken was assessed 4 times before and after treatment as follows: (i) before treatment control (Pre-T1); (ii) 30-min post ivermectin treatment (0.5 h Post-T1); (iii) 2 days post treatment (2 days Post-T1); and (iv) 7 days post treatment (7 days Post-T1). Mortality of bed bugs in each replicate was assessed every 24 h up to 7 days (168 h) post treatment.

#### Fluralaner

Twenty-one birds from the flock of 30 birds were randomly assigned to control and treatment groups. Some birds used in the ivermectin experiments were included in this experiment; these birds were used at least 1 month after the completion of the ivermectin experiment. Since no fluralaner-containing products are labeled in the USA for use in poultry, we used an oral fluralaner formulation licensed for use in domestic dogs (Bravecto®; Merck Animal Health, Rahway, NJ, USA). Bravecto® was administered topically at a dose of 2.5 mg/kg to the featherless lateral aspect of the neck of each chicken. We used two doses for oral administration of Bravecto®: (i) in experiment 1, 2.5 mg/kg body mass was administered once, representing the high dose treatment; (ii) and in experiment 2, 0.5 mg/kg was administered at baseline (day 0; treatment 1 [T1]) and again 7 days later (day 7; treatment 2 [T2]), representing the lower dose treatment. The high dose was based on Prohaczik et al. [26], whereas the lower dose regime represented a protocol approved by regulatory authorities in Australia and the European Union for use of a fluralaner-containing product on chickens (Exzolt®; MSD Animal Health, Munich, Germany) [27]. The target doses of fluralaner were obtained by weighing portions of Bravecto® tablets, as appropriate for each weighed bird. Chickens that received the high dose (2.5 mg/kg, experiment 1) were assessed 7 times, as follows: (i) before treatment control (Pre-T1); (ii) 30 min post fluralaner administration (0.5 h Post-T1); (iii) 2 days (2 days Post-T1); (iv) 7 days (7 days Post-T1); (v) 14 days (14 days Post-T1); (vi) 21 days (21 days Post-T1); and (vii) 28 days post initial treatment (28 days Post-T1). Birds that received the lower dose of fluralaner (0.5 mg/kg, experiment 2) were assessed an additional 2 times (9 times in total), including 30 min after the administration of the second dose on day 7 (7 days Post-T1 = 0.5 h Post-T2) and 2 days later (9 days Post-T1 = 2 days Post-T2). Mortality of bed bugs in each group was assessed every 24 h for 7 days post blood-feeding.

### Data analysis

The fluralaner LC_50_, LC_90_ and LC_99_ (lethal doses 50%, 90%, 99%, respectively) for each bed bug population were determined using log-dose probit-mortality analysis based on a spreadsheet template [28]. The values agreed with the results of the analysis in PoloPlus (LeOra Software, Petaluma, CA, USA). Abbott’s correction [29] was used to correct for control mortality, as needed. The fluralaner dose–response curve of each bed bug population was compared to that of the insecticide-susceptible Harlan bed bug population. Likewise, log-dose probit-mortality analysis of Harlan strain bed bugs was used to obtain an estimate of the LD_99_ value for deltamethrin. This dose was used as a diagnostic dose on the five field-collected strains. In vitro toxicity of fluralaner to each population was compared to that of the insecticide-susceptible Harlan population using a resistance ratio (RR), calculated as: (LC_50_ of the field-collected population)/(LC_50_ of the Harlan population). We used the lethal dose ratio significance test: the 95% confidence limits of the RR were calculated, and if this confidence interval (CI) did not include the value of 1.0, then the RR at the LC_50_ was considered to be significant [30]. The effects of ivermectin and fluralaner treatments on Harlan bed bugs that fed on the treated birds were determined over time using linear mixed model with repeated measures (based on restricted maximum likelihood [REML]) and Tukey’s honestly significant difference (HSD) test [31]. Means are presented with standard error of the mean. Differences between the two dose treatments of fluralaner in vivo at each time point post treatment were detected using Student’s t-test [31].

## Results

### Pyrethroid resistance

Deltamethrin was topically applied to Harlan strain bed bugs and a probit analysis of their log-dose–response was conducted. The LD_50_ and LD_90_ were 1.4 and 4.0 ng/male, respectively (Table 1). The estimated LD_99_ was 11.8 ng deltamethrin, and this dose was used as a diagnostic dose that was topically applied to male bed bugs from the five field-collected populations. We found low mortality in all field-collected strains of bed bugs, including strain WS that we collected in 2008 (Table 2), indicating a high resistance to deltamethrin.

**Table 1:** Dose-mortality assays using deltamethrin with adult male bed bugs from an insecticide-susceptible (Harlan) population of *Cimex* *lectularius*

Active ingredient	*n*	LD_50_, ng (95% CI)^a^	LD_90_, ng (95% CI)^a^	LD_99_, ng (95% CI)^a^	Slope ± SEM	*χ*^2^ (*df*)	t-ratio^b^
Deltamethrin	140	1.4 (1.0, 2.1)	4.0 (2.8, 7.1)	11.8 (5.9, 51.5)	2.5 ± 0.3	5.8 (5)	7.2*

*CI* Confidence interval,* LD*_*50*_ lethal dose that killed 50% of bed bugs,* LD*_*90*_ lethal dose that killed 90% of bed bugs,* LD*_*99*_ lethal dose that killed 99% of bed bugs, SEM standard error of the mean

^a^LD_50_, LD_90_, LD_99_ were estimated from probit analysis. Values are given in nanograms per insect

^b^t-Ratio of the slope. Values > 1.96 denote a significant regression at *P* < 0.05

**Table 2:** Fluralaner dose-mortality assays, resistance ratios and deltamethrin diagnostic dose assays of five recently collected *C.* *lectularius* populations relative to an insecticide-susceptible (Harlan) population

Population (abbreviation), year collected	Collection location (USA)	Fluralaner^a^							Deltamethrin
		*n*	LC_50_, ng/ml (95% CI)^b^	LC_90_, ng/ml (95% CI)^b^	Slope ± SEM	*χ*^2^ (*df*)	*t*-Ratio^c^	RR at LC_50_^d^ (95% CI)	% mortality in HA at LD_99_ (*n*)^e^
Harlan (HA), 1973 (insecticide susceptible)	Fort Dix, NJ	237	15.3 (11.7, 19.8)	38.6 (28.3, 71.9)	3.2 ± 0.4	7.9 (5)	8.0*	N/A	N/A
Cincinnati (CIN), 2012	Cincinnati, OH	219	18.4 (16.2, 23.5)	51.7 (41.4, 72.5)	2.8 ± 0.3	3.5 (6)	8.2*	1.2	13 (45)
Fuller Mill (FM)^f^, 2017	High Point, NC	202	23.2 (13.8, 35.3)	67.5 (42.5, 216.6)	2.8 ± 0.3	12.6 (5)	7.8*	1.5*	5 (40)
Winston Salem (WS), 2008	Winston Salem, NC	226	22.0 (15.3, 30.6)	92.7 (58.4, 230.8)	2.0 ± 0.3	8.0 (6)	7.4*	1.4*	0 (48)
Shanda (SH), 2017	Raleigh, NC	220	21.1 (17.5, 25.4)	65.0 (48.7, 102.0)	2.6 ± 0.3	1.3 (5)	7.7*	1.4*	0 (46)
Poultry House (PH), 2021	Pennsylvania	212	18.0 (15.3, 22.1)	43.6 (34.7, 61.6)	3.3 ± 0.4	4.9 (5)	7.8*	1.2	2 (50)

		Ivermectin^a^							
Harlan (HA), 1973 (insecticide susceptible)	Fort Dix, NJ	231	61.0 (52.7, 69.9)	114.9 (95.4, 154.7)	4.7 ± 0.7	3.0 (4)	6.4*	N/A	N/A

Ivermectin dose-mortality assays are also shown for the Harlan (HA) population

*N/A* Not available,* RR* resistance ration

^a^Only fully fed adult male bed bugs were included in these assays

^b^LC_50_ or LC_90_ of the bed bugs, estimated from probit analysis for each population. Values are in ng/ml of human blood

^c^*t*-Ratio of the slope. Values > 1.96 denote a significant regression at *P* < 0.05

^d^The RR was calculated as LC_50_ of field-collected strain/LC_50_ of susceptible reference strain (HA). RR values with asterisk (*) were considered to be significant when their 95% CIs did not include 1.0 [31]

^e^The LD_99_ dose of deltamethrin was estimated for the HA population from log dose–response probit analysis. This dose was used as a diagnostic dose and topically applied to field-collected bed bugs. Deltamethrin was diluted in acetone and 0.5 µl was applied to each insect. Percentage mortality at 2 days post administration and (*n*) are reported

^f^Fuller Mill is the same strain referred to as Fuller Miller in González-Morales et al. [41]

### In vitro feeding assays using artificial feeders

**Fig. 3:**
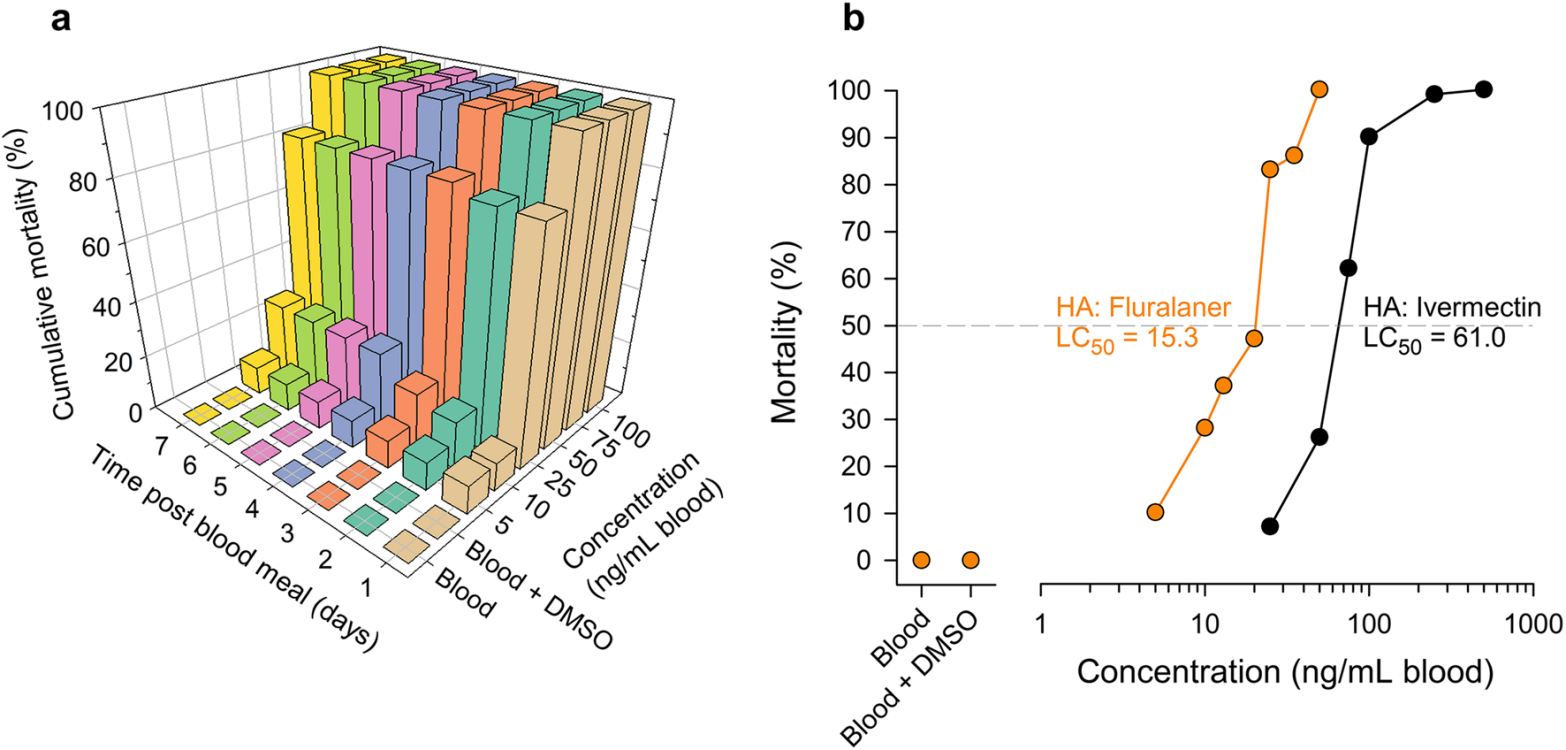
In vitro dose–response curves for bed bugs. **a** Three-dimensional dose–response and time-course representation of mortality of the insecticide-susceptible HA adult male bed bugs fed fluralaner-supplemented human blood. Fully fed bed bugs were monitored for 7 days. **b** In vitro fluralaner and ivermectin log dose–response curves for HA adult male bed bugs. Fluralaner and ivermectin were separately dissolved in DMSO and mixed with human blood to obtain various concentrations of insecticides in 0.1% DMSO in blood. Blood and Blood + DMSO represent the control treatments: there was no mortality in control bed bugs. Mortality at 7 days post-ingestion of chicken blood by bed bugs is reported. At least three replicates of 10 adult male bed bugs per replicate were performed per concentration. The LC_50_ estimates were based on probit analyses. DMSO, Dimethyl sulfoxide; HA, Harlan strain bed bugs; LD_50_, lethal dose that killed 50% of bed bugs

A comparative dose–response analysis using technical grade fluralaner and ivermectin, each dissolved in DMSO and added to human blood, was conducted using an artificial feeding system. In this time-course study we evaluated daily mortality of the insecticide-susceptible bed bugs (reference) fed various concentrations of fluralaner and ivermectin. The relationship between cumulative mortality, concentration of fluralaner in blood and time since bed bugs fed (range: 0–7 days) is shown in Fig. 3a. Based on the results of this analysis, we chose to evaluate mortality 7 days after bed bugs fed on medicated human blood. The results from the in vitro feeding experiments are shown in Table 2 and Fig. 3b. The LC_50_ value of fluralaner, tested on adult males of insecticide-susceptible Harlan bed bugs, was 15.3 ng/ml blood (95% CI: 11.7, 19.8 ng), and the estimated LC_90_ value was 38.6 ng/ml blood. We conducted a similar dose–response study using technical grade ivermectin and adult male bed bugs of the Harlan strain. The LC_50_ value was 61.0 ng/ml (95% CI: 52.7, 69.9 ng) and the estimated LC_90_ value was 114.9 ng/ml blood (Table 2; Fig. 3b).

**Fig. 4:**
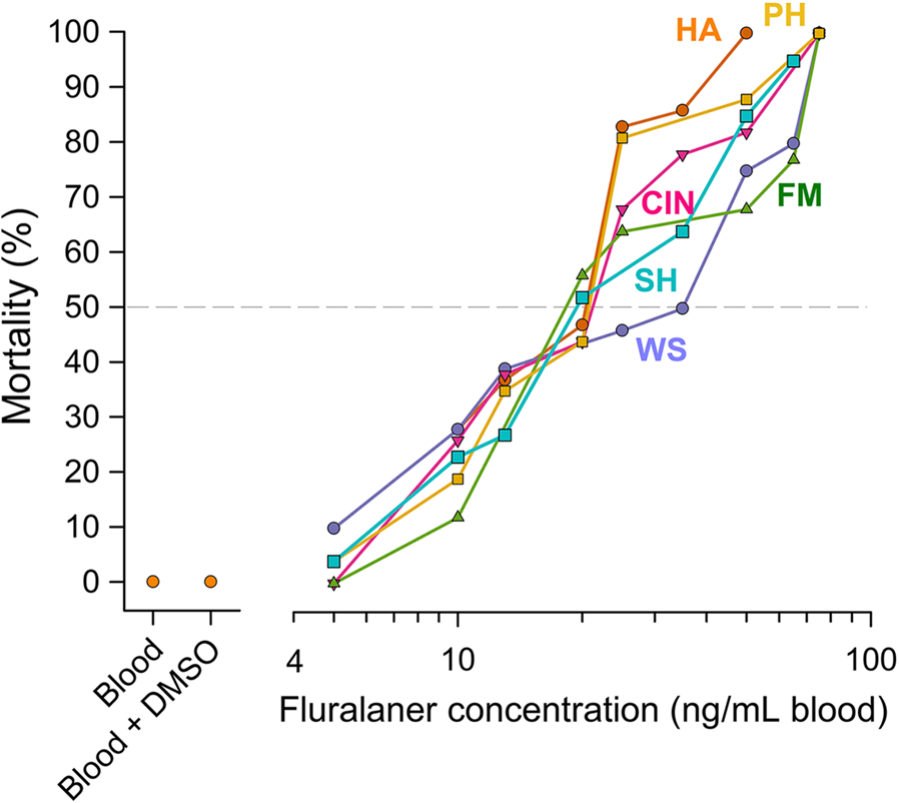
In vitro fluralaner log dose–response curves for male bed bugs from six populations, including five field-collected strains and the reference insecticide-susceptible strain (HA). Fluralaner was dissolved in DMSO. See Table 2 for abbreviations of bed bug strains

The fluralaner LC_50_ values of the five field-collected bed bug strains ranged from 18.0 to 23.2 ng/ml blood (Table 2; Fig. 4); these values did not differ significantly from those of the Harlan strain bed bugs based on overlap of their respective 95% CIs. Although some significant insecticide resistance was found when the Fuller Mill, Winston Salem and Shanda bed bugs were compared with those of the Harlan insecticide-susceptible population, the low resistance ratios (based on LD_50_ values) of 1.2–1.5 indicate minimal or no resistance to fluralaner. Further, the relatively steep slopes of all dose–response curves in all bed bug strains are indicative of homogeneous populations in their responses to the ingestion of fluralaner. No evidence of any correlation between deltamethrin-caused mortality and fluralaner RR across the five strains was found (Spearman’s rank correlation coefficient [rs] = 0.2294, *P* = 0.7105; *n* = 5). We did not evaluate the effect of ivermectin on the field-collected strains because the administration of ivermectin to chickens was ineffective at killing bed bugs (see following section).

### In vivo feeding assays with chickens

At the dose of ivermectin administered to chickens (0.2 mg/kg), treatments via injection did not result in any mortality in bed bugs that fed on the medicated chickens. However, when the same dose of ivermectin was administered via oral gavage, 5 ± 2.4% (range: 0–14%; *n* = 6 chickens) of the bed bugs that fully fed (5–11 of 15 bed bugs per replicate) on chicken blood 2 days after chickens were treated died. Although this low mortality was significantly higher (*P* < 0.05) than that at baseline (day 0, before ivermectin was administered to chickens), because none of the bed bugs that fed on ivermectin-treated chickens 7 days post treatment died, further studies with ivermectin were discontinued.

**Fig. 5:**
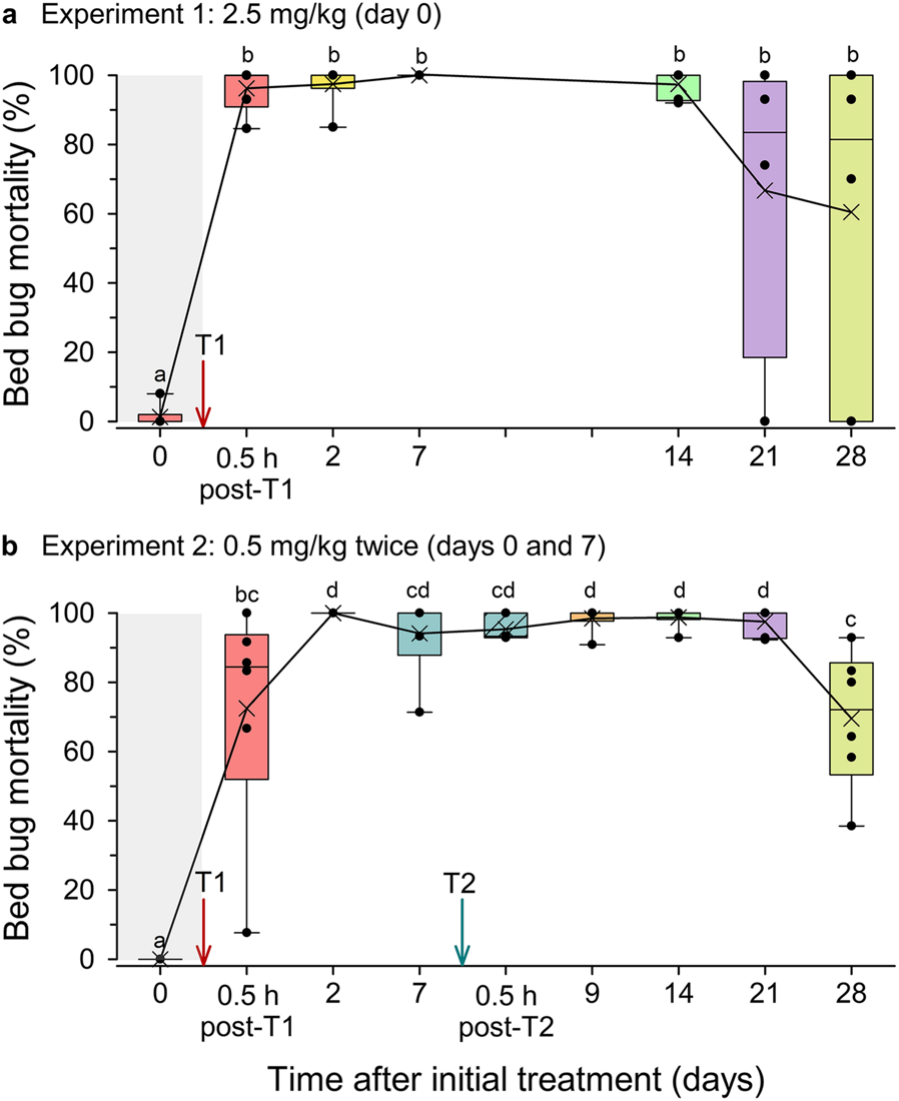
In vivo assays with chickens treated with fluralaner by oral gavage. **a** Experiment 1: chickens treated with 2.5 mg/kg body mass on day 0. **b** Experiment 2: Chickens treated with 0.5 mg/kg body mass on day 0 and again on day 7. Each experiment consisted of 6 birds. A maximum of 15 bed bugs were fed on each bird at each time point, with each time point therefore represented by 78–87 bed bugs (out of a maximum of 90 bed bugs) that fed to repletion. A linear mixed model (based on restricted maximum likelihood) was conducted within each experiment followed by Tukey’s honestly significant difference test to separate means (represented within box plots by X). Means with different lowercase letters (above box plots) are significantly different at *P* < 0.05. T1, Treatment 1

**Fig. 6:**
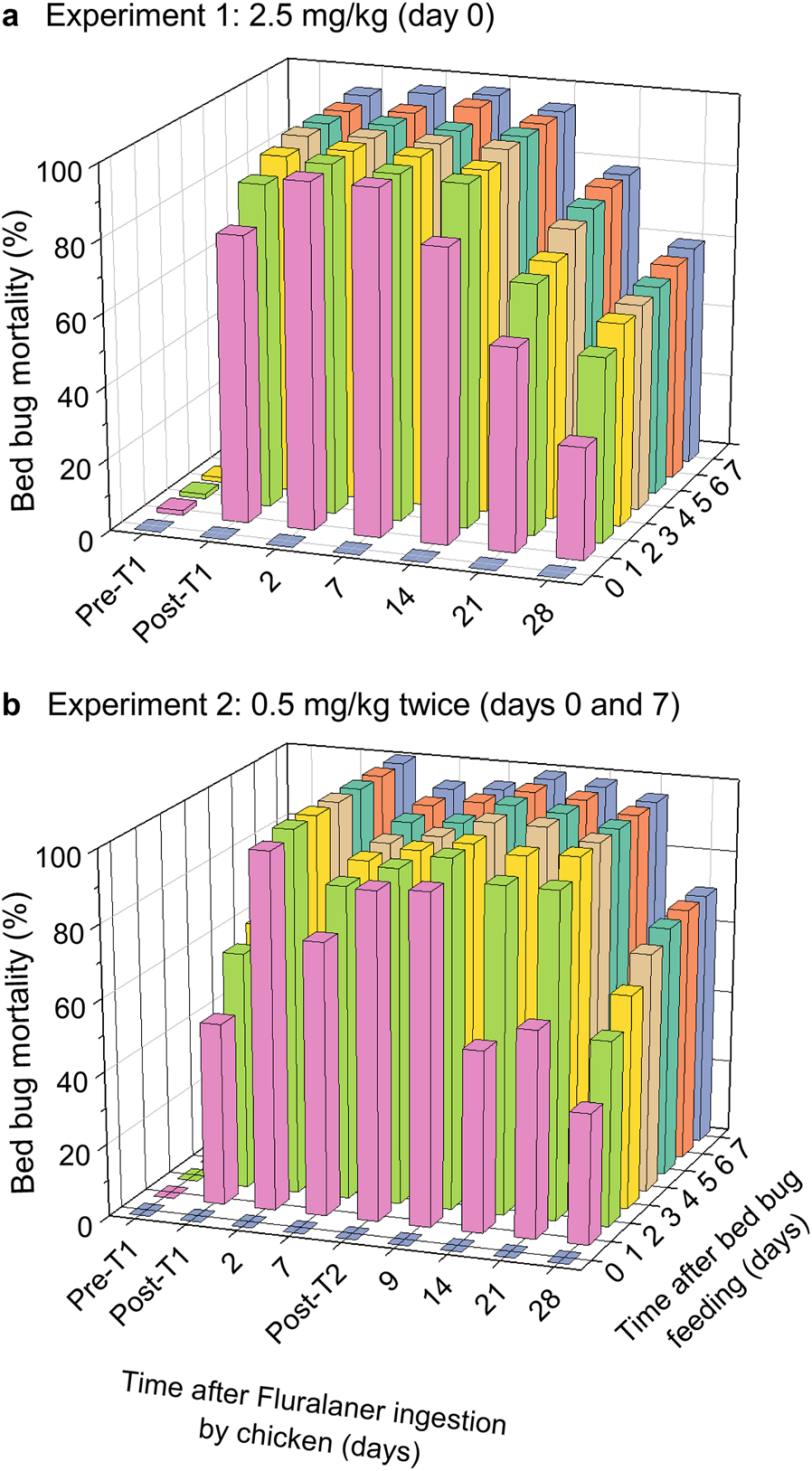
Three-dimensional representation of the in vivo assays shown in Fig. 5. In addition to the cumulative mortality on day 7 after initial treatment (shown in Fig. 5), the time-course of mortality of HA strain bed bugs is shown on days 1–7 after they fed on treated birds. **a** Experiment 1: chickens treated with 2.5 mg/kg body mass on day 0. **b** Experiment 2: chickens treated with 0.5 mg/kg body mass on day 0 and again on day 7. Each experiment included 6 birds. At each time point a maximum of 15 bed bugs were fed on each bird, with each time point therefore represented by 78–87 bed bugs (out of a maximum of 90 bed bugs) that fed to repletion

Two experiments with the aim to assess the efficacy of fluralaner in chickens against bed bugs were performed (Fig. 5). For each experiment we developed a time-course of mortality (days 0 to 28), and we monitored bed bugs daily for 7 days after they fully fed on chicken blood (Fig. 6). Quantitative analysis was based on cumulative percentage mortality on day 7, at which time both treatment bed bug groups showed a high mortality and control bed bugs that had fully fed on untreated chickens showed an overall low mortality (< 1.3%) (Fig. 6a, b).

In experiment 1 (2.5 mg fluralaner/kg), 96.3 ± 2.6% (range: 84.6–100%; *n* = 6 chickens) of the bed bugs that fully fed (5–11 of 15 bed bugs per replicate) on chicken blood 0.5 h post gavage treatment died (Fig. 5a). Mortality was significantly higher at all time points post fluralaner treatment (0.5 h to 28 days) than before treatment on day 0 (1.3 ± 1.3%) (linear mixed model, *F* = 14.2281, *df* = 8, *P* < 0.0001; Tukey’s HSD). Mean mortality peaked on day 7 (100%) and remained > 97% up to day 14. There was higher variation and an overall decline in bed bug mortality 21 days (66.8 ± 22.9%, range: 0–100%) and 28 days (60.5 ± 19.6%, range: 0–100%) post fluralaner treatment. It should be noted that on day 21, we assayed only four of the six chickens due to technical constraints. Nevertheless, there were no significant differences in bed bug mortality across all time points post fluralaner gavage treatment (*P* > 0.05) (Fig. 5a). A graphical representation of the time-course of bed bug mortality before and after the chickens were fed fluralaner (days 0–28) and before and after bed bugs fully fed on chicken blood (days 0–7) is shown in Fig. 6a.

In Experiment 2, six chickens of the same flock as in experiment 1 were treated by gavage with 0.5 mg fluralaner/kg on day 0 and again on day 7. The overall pattern of bed bug mortality was similar to that in experiment 1 (Fig. 5b). Mortality was significantly higher at all post gavage time points than on day 0 (0% mortality before the gavage administration) (linear mixed model, *F* = 38.8355, *df* = 8, *P* < 0.0001; Tukey’s HSD). However, bed bugs fed on chickens 0.5 h after the first treatment had lower mortality (72.5 ± 13.7%, *P* = 0.0296; *n* = 6 chickens; Tukey’s HSD) than mean bed bug mortality 2 days post treatment (100%); there was also greater variation across the six replicates (range: 7.7–100%) 0.5 h after the first treatment. Bed bug mortality remained > 95% up to 21 days post treatment and there were no significant differences in mortality between days 2 and 21 (Tukey’s HSD, *P* > 0.05). By day 28, however, mean mortality significantly declined to 69.5 ± 8.1% (*P* < 0.05), and we observed higher variation among replicates (range: 38.5–92.9%) (Figs. 5b, 6b). It should again be noted that in this experiment, a second gavage treatment with 0.5 mg fluralaner/kg was administered on day 7; a graphical representation of the time-course of bed bug mortality before and after the chickens were fed fluralaner (days 0–28) and before and after bed bugs fully fed on chicken blood (days 0–7) is shown in Fig. 6b.

The results of experiments 1 and 2, by day, were compared after the first gavage treatment. There were no significant differences between the results in experiment 1 and those in experiment 2 at any time point after the initial treatment (t-tests, *P*-value range: from 0.1199 on day 2 to 0.6792 on day 28). It is important to note, however, that at the termination of both experiments on day 28, variation in bed bug mortality across replicates was higher in experiment 1 (high dose administered once; range: 0–100%) than in experiment 2 (range: 38.5–92.9%).

## Discussion

This is the first study to explore the systemic use of veterinary drugs (xenointoxication) to control bed bugs as ectoparasites of chickens. Unlike holometabolous blood-feeders (e.g. mosquitoes), all developmental stages of bed bugs must obtain a blood meal from a host to develop and reproduce. Moreover, both male and female bed bugs are obligatorily dependent on blood meals, unlike male mosquitoes which feed on nectar and not on blood. Hematophagy in all mobile stages of bed bugs makes systemic antiparasitic drugs especially appropriate for consideration in bed bug management.

### Use of Ivermectin to control bed bugs

In the present study, the LC_50_ for bed bugs was 61.0 ng/ml, similar to that reported with ivermectin-supplemented heparinized mouse blood [32]. Therefore, a blood concentration > 61.0 ng/ml, maintained for several days, would be desirable for the effective suppression and ultimately elimination of bed bugs in chicken facilities. However, multiple bioassays with bed bugs and pharmacokinetic studies in chickens suggest that ivermectin does not reach this target concentration in blood. For example, administration of ivermectin to laying hens by the ingestion route, at 0.2 mg/kg, resulted in ivermectin rapidly reaching a maximum concentration (*C*_max_) of only 10.2 ng/ml at 3.4 h post treatment, followed by a rapid decline, with an elimination half-life of only 0.23 days [25]. Similar results were reported following the administration of ivermectin to broiler chickens at 0.4 mg/kg in drinking water on two consecutive days, and again 14 days later; although ivermectin reached maximum plasma concentrations of 145.5–182.7 ng/ml within 30–60 min post treatment, it rapidly declined to undetectable levels by 12–24 h post treatment [33]. When ivermectin was injected intravenously at 0.2 mg/kg body mass, *C*_max_ reached 316.0 ng/ml 6 h later, but it fell below the target concentration for bed bugs in < 1 day [25]. Finally, in a recent evaluation of the effects of ivermectin-treated backyard hens on *Culex* mosquitoes, chickens were fed ivermectin-supplemented feed for 72 consecutive days (200 mg ivermectin/kg feed and 0.151 kg feed/chicken daily) [34], representing a very high dose of 30.2 mg ivermectin per chicken per day. However, plasma concentrations in the treated chickens averaged only 33.1 (range: < 5–155.2) ng/ml, and they peaked early in the study (54.9 ng/ml on day 11) and declined to much lower concentrations over the 72-day-long study (20.6 ng/ml on day 70) [34]. Overall, these studies consistently show low bioavailability of ivermectin in chicken blood, likely due to rapid detoxification and clearance from the blood and possibly other traits, such as high metabolic rate [25]. Therefore, notwithstanding the sublethal effects of ivermectin on bed bugs (morbidity, including lower fecundity, difficulty feeding and incomplete molts) [35], we tentatively conclude that treatments with ivermectin might not be effective for the elimination of bed bugs from infested poultry farms.

### Use of fluralaner to control bed bugs

Fluralaner is an isoxazoline (IRAC group 30) that has been extensively tested as a systemic insecticide against ectoparasitic insects, ticks and mites, mainly on dogs and cats, but also on livestock and zoo and feral animals. Bravecto® (containing racemic fluralaner) is labeled for dogs and cats, and because of its long elimination half-life, it is administered every 3 months [36]. This unique property of fluralaner contrasts with ivermectin and prompted us to examine its effects on bed bugs. However, because there are no fluralaner-containing products labeled for use in chickens in the USA, we used Bravecto® but experimentally followed the dosage directions for Exzolt®, a racemic fluralaner-containing product approved in Australia and the European Union to control poultry ectoparasites, such as the poultry red mite and the northern fowl mite [27]. The 1% aqueous formulation of this product is designed as a drinking water treatment to be administered twice at a dose of 0.5 mg/kg, 7 days apart, and provides up to 3 months of effective mite control [27, 37]. The high efficacy of this approach in controlling mites on poultry [22] and its negligible health effects, as well as high safety to birds [26], make fluralaner particularly attractive for testing with bed bugs.

We first conducted a dose–response study with insecticide-susceptible bed bugs feeding on fluralaner-supplemented human blood using an artificial membrane-based feeding system. Our results showed LC_50_ and LC_90_ values of 15.3 and 38.6 ng fluralaner/ml blood, respectively. In a previous study with bed bugs fed fluralaner-supplemented sheep blood, high levels of bed bug incapacitation, defined as death or immobility, were observed in various life stages at ≥ 100 ng fluralaner/ml blood [15].

Next, we conducted a proof-of-principle experiment with a single high dose of Bravecto® (2.5 mg/kg), and then a second experiment, adjusting the Bravecto® dose to match the Exzolt® label dose (0.5 mg/kg, administered twice, 7 days apart). It is important to note, however, that in all these experiments fluralaner was administered orally (by gavage) in tablet form using weighed portions of Bravecto® tablets, whereas Exzolt® is delivered in drinking water. In both experiments, a single blood meal on medicated chickens resulted in high bed bug mortality even at 28 days after the chickens were treated with fluralaner.

Our results suggest that the Bravecto® treatments might be substantially less effective than those expected with the Exzolt® treatments. In a pharmacokinetic study involving the oral administration of Exzolt® to laying hens (0.5 mg/kg, administered twice, 7 days apart), high plasma concentrations were reached, with a *C*_max_ of 323.7 ng/ml at 36 h after the first treatment and 355.1 ng/ml at 12 h after the second treatment on day 7 [27]. Given an elimination half-life of fluralaner in chicken plasma of about 5 days [27], the concentration of this drug in blood is expected to decline to approximately 44 ng/ml 22 days after the first administration of fluralaner, which is still above the concentration required to kill 90% of the bed bugs. Thus, the high plasma concentrations of fluralaner obtained in previous studies with Exzolt® would be expected to kill 100% of the bed bugs for at least the first 14 days post initial treatment. Likewise, in a previous study, treatments of hens with a liquid formulation of fluralaner killed 100% of the poultry red mite for up to the first 15 days post treatment [21], and resulted in up to 93% reduction of the mite population even 70 days post treatment [38]. These high mortality and high control rates, coupled with the mites’ LC_50_ of approximately 125 ng/ml blood [37, 39], again suggest that Exzolt® delivers high plasma concentrations of fluralaner. The much lower LC_50_ of bed bugs than poultry red mites would predict a much better efficacy of fluralaner on bed bugs than on mites. These suppositions need to be tested empirically with in vivo treatments with Exzolt® and pharmacokinetic studies.

All of the field-collected bed bug strains that we tested showed a high resistance to deltamethrin, a pyrethroid commonly used to manage bed bug infestations. However, bed bugs from these strains were highly susceptible to fluralaner. The high efficacy of fluralaner on pyrethroid-resistant bed bugs is particularly important in the poultry industry because most of the products labeled to manage bed bug populations in the farm environment contain pyrethroid insecticides. Our findings are consistent with results showing a higher performance of fluralaner than deltamethrin against a wide range of arthropod pests, including *Stomoxys* *calcitrans* (stable fly), *Rhipicephalus* *sanguineus* (brown dog tick), *Aedes* *aegypti* larvae (yellow fever mosquito) and *Lucilia* *cuprina* (Australian sheep blowfly) [40].

Fluralaner, an isoxazoline (IRAC class 30), has insecticidal and acaricidal activity and a dual mode of action as inhibitor of the gamma-aminobutyric acid-gated chloride channels (GABACl) and L-glutamate-gated chloride channels (GluCls) [40]. Fipronil, a phenylpyrazole (IRAC class 2B), also targets these channels and is commonly used to control household insect pests. Insecticide resistance to fipronil involves metabolic detoxification and target-site insensitivity associated with a specific mutation in the *Rdl* gene that results in A302S amino acid substitution; this substitution also confers high resistance to dieldrin, a cyclodiene (IRAC class 2A) [40]. In a previous study, the same field-collected bed bug strains used in the present study exhibited variable, but high resistance to fipronil (4.4- to > 492-fold) [41]. However, none of these bed bugs had the mutation in the *Rdl* gene associated with resistance to fipronil and dieldrin. Moreover, Gassel et al. [40] showed that fluralaner efficacy is unaffected by dieldrin and fipronil resistance in the cat flea, ticks and fruit fly, indicating a lack of cross-resistance due to fluralaner targeting a site on GABACl channels distinct from the site targeted by cyclodienes and fipronil. These findings suggest that cross-resistance to fipronil and dieldrin is not likely to interfere with the efficacy of fluralaner on bed bugs in poultry farms. Nevertheless, consideration of fluralaner for bed bug control should proceed with caution because bed bug populations may be experiencing selection with fluralaner and afoxalaner through ongoing exposure to Bravecto®- and NexGard®-medicated dogs and cats.

### Study limitations

Foremost, it is important to reiterate that there are no fluralaner-containing products labeled for use on chickens in the USA. Therefore, we used weighed portions of Bravecto®, labeled for use in dogs, and experimentally followed the dosage directions for Exzolt®, a fluralaner-containing product approved in Australia and the European Union for use on chickens. This extra-label use of Bravecto® is allowed in accordance with the Animal Medicinal Drug Use and Clarification Act, provided there are no violative drug residues [42]. Our use of Bravecto® in hens was strictly experimental and is not meant to condone its use with commercial flocks.

We used only adult male bed bugs. Biosafety concerns related to transporting bed bugs between two laboratories 3.5 km apart precluded the use of nymphs and adult females. Although previous studies with membrane-based artificial feeders showed no major differences among different life stages of bed bugs [13], follow-up studies should include nymphs and females. Also, we tested only five field-collected strains from the eastern USA. Many more field-collected bed bug populations need to be sampled to determine whether our results are broadly applicable across the USA and globally.

Finally, our study was conducted in controlled laboratory environments. Drug treatments, metabolic rates, bed bug behavior and clearance rates of drugs are all bound to vary under field conditions. Therefore, studies with commercial chicken flocks under field conditions are warranted.

## Conclusions

This is the first report of a novel management strategy to control bed bug infestations in poultry farms. The administration of fluralaner tablets by oral gavage, at a dose of 0.5 mg/kg chicken body weight, repeated 7 days after the first treatment, was highly effective at killing bed bugs for the first 28 days post treatment. Based on its pharmacokinetic parameters in chickens, similar dosing of fluralaner in drinking water is expected to be even more effective against bed bugs. A combination of monitoring, education, heat treatments and xenointoxication could hold the key to eradicating bed bugs from infested poultry farms.

## Data Availability

The data sets supporting the results are available from CS (coby@ncsu.edu) upon reasonable request.

## Abbreviations

AI: Active ingredient
CI: Confidence interval
*C*_max_: Maximum plasma concentration
DMSO: Dimethyl sulfoxide
LC_50_: Lethal concentration that killed 50% of the population
LC_90_: Lethal concentration that killed 90% of the population
LC_99_: Lethal concentration that killed 99% of the population
*Rdl*: Resistant to dieldrin
RR: Resistance ratio
